# Sulforaphane Attenuates Antipsychotic-Elicited Hypercholesterolemia Through Inhibition of Pregnane X Receptor

**DOI:** 10.3390/biom16101455

**Published:** 2026-10-06

**Authors:** Jianfei Pan, Zhaojie Meng, Seung Hyun Park, Hong Chen, Shuxia Wang, Changcheng Zhou

**Affiliations:** 1 Division of Biomedical Sciences, School of Medicine, University of California, 900 University Avenue, Riverside, CA 92521, USA; 2 Vascular Biology Program, Boston Children’s Hospital and Harvard Medical School, Boston, MA 02115, USA; 3 Department of Pharmacology and Nutritional Science, University of Kentucky, Lexington, KY 40536, USA

**Keywords:** antipsychotics, olanzapine, sulforaphane, PXR, hyperlipidemia, cardiovascular disease

## Abstract

Olanzapine is a widely prescribed antipsychotic that has been associated with increased risk of cardiometabolic disorders including hyperlipidemia. However, the underlying mechanisms of olanzapine’s dyslipidemic properties are poorly understood. We previously identified several clinically used drugs with dyslipidemic properties as ligands for the nuclear receptor pregnane X receptor (PXR), a key regulator of xenobiotic metabolism. Recent studies have revealed the important functions of PXR in lipid homeostasis. Here, we report that olanzapine is a potent PXR agonist that elicits hyperlipidemia through PXR signaling. Olanzapine activated PXR to stimulate key intestinal lipogenic gene expression in primary cells and in vivo. Exposure to olanzapine caused higher plasma total cholesterol and atherogenic very low-density lipoprotein (VLDL), as well as low-density lipoprotein (LDL) cholesterol levels, in mice in a PXR-dependent manner. We then explored the therapeutic potential of a previously identified human PXR antagonist, sulforaphane, and found that sulforaphane can efficiently block olanzapine-elicited PXR activation and attenuate olanzapine-elicited hypercholesterolemia in PXR-humanized mice. Our studies provide mechanistic insights for understanding the cardiovascular disease risk associated with antipsychotic olanzapine treatment in patients and demonstrate the therapeutic potential of PXR antagonists in mitigating the adverse effects of PXR agonistic drugs on cardiovascular health.

## 1. Introduction

Schizophrenia is one of the most common, burdensome, and costly psychiatric disorders in adults worldwide, affecting nearly 1% of the world’s population, and is one of the top ten causes of disability in the world [1,2,3]. Currently, atypical antipsychotic drugs (AAPDs) are its primary treatment of choice [4]. Olanzapine is a highly efficacious second-generation atypical antipsychotic agent frequently prescribed for the treatment of schizophrenia, bipolar disorder, major depressive disorder as adjunctive treatment, and agitation associated with schizophrenia or bipolar disorder since its initial approval by the US Food and Drug Administration in 1996 [5]. Despite its clinical efficacy and widespread use, concerns have been raised regarding its tolerability and safety profile, particularly in relation to metabolic adverse effects [6]. The administration of olanzapine has been linked to several metabolic abnormalities, including weight gain, hyperlipidemia, blood glucose abnormalities, insulin resistance, and hypertension [7,8,9,10,11]. These metabolic disturbances increase the risk of type 2 diabetes, cancer, and cardiovascular disorders, ultimately impairing the quality of life of patients with schizophrenia. However, the underlying mechanisms of olanzapine-induced metabolic abnormalities remain unclear.

We previously identified several clinically used drugs with dyslipidemic properties, including an atypical antipsychotic, quetiapine, as potent agonists for the nuclear receptor pregnane X receptor (PXR) [12,13]. PXR, also known as steroid and xenobiotic receptor (SXR), is a unique nuclear receptor that can be activated by many chemicals, including pharmaceutical agents, environmental chemicals, and endogenous hormones [13,14,15,16]. PXR functions as a xenobiotic sensor that regulates the expression of genes required for xenobiotic metabolism in the liver and intestine, including cytochrome P450 (CYP) enzymes (e.g., CYP3A4), conjugating enzymes (e.g., glutathione transferase [GST]), and ABC family transporters (e.g., multidrug resistance 1 [MDR1]) [13,14]. In addition to xenobiotic metabolism, we and others recently revealed the important function of PXR in regulating lipid metabolism and atherogenesis [12,13]. While PXR can regulate many hepatic and intestinal genes mediating lipid homeostasis [13,17,18,19,20,21,22,23,24], the functions of PXR in mediating the adverse effects of certain clinically used drugs on lipid homeostasis are still poorly understood.

In this study, we investigated the potential contribution of PXR signaling to olanzapine’s dyslipidemic effects in vitro and in vivo. In addition, we also explored the therapeutic potential of a naturally derived PXR antagonist, sulforaphane [25], in preventing olanzapine-induced dyslipidemia. We found that olanzapine is a potent human PXR agonist that induces hyperlipidemia through PXR signaling. Furthermore, we demonstrate that sulforaphane can efficiently inhibit olanzapine-mediated PXR activation and dyslipidemia, which provides a potential therapeutic strategy to mitigate the adverse consequences of olanzapine treatment on cardiovascular health.

## 2. Materials and Methods

### 2.1. Animals and Treatments

PXR-humanized (hPXR•mPXR^-/-^) and PXR-null (mPXR^-/-^) mice on a C57BL/6 background have been described before [26,27]. The hPXR•mPXR^-/-^ and mPXR^-/-^ mice littermates used in the animal experiment had the same mPXR-deficient background, and the hPXR•mPXR^-/-^ mice carried the single allele containing the human PXR gene. For the ligand treatment, 8-week-old male hPXR•mPXR^-/-^ and control mPXR^-/-^ littermates were fed *ad libitum* on a semisynthetic low-fat AIN76 diet (4.2% fat and 0.02% cholesterol; Research Diet) [12,26] and administered vehicle control (corn oil), olanzapine (10 mg/kg body weight/day), or a combination of olanzapine and sulforaphane (10 mg/kg body weight/day) for 7 days. Our selected dose and treatment duration was based on the interspecies scaling factor of 12.3 between mice and humans [28,29] and previous studies on PXR ligand-induced dyslipidemia [12,26]. On the day of euthanasia, mice were fasted for 6 h following the dark cycle (feeding cycle), and blood and tissues were collected [12,26]. All animal studies were performed in compliance with the protocols approved by the Institutional Animal Care and Use Committees of the University of California, Riverside, and the University of Kentucky.

### 2.2. Reagents and Plasmids

Olanzapine (O1141) and quetiapine (PHR1856) were purchased from Millipore Sigma (St. Louis, MO, USA), and sulforaphane (10496) was purchased from Cayman Chemical (Ann Arbor, MI, USA). All the chemicals were dissolved in DMSO. All the plasmids used in this study have been described before [12,26], including human (h) and mouse (m) PXR expression vectors, GAL4 DNA-binding domain–linked (DBD-linked) nuclear receptor LBD vectors, VP16-hPXR, GAL4 DBD–linked nuclear receptor coregulators (SRC-1 and PBP), the PXR-dependent CYP3A4 promoter reporter (CYP3A4 XREM-luciferase), the GAL4 reporter (MH100-luciferase), and the CMX–β-galactosidase expression vector [12,26].

### 2.3. Cell Culture and Transfection Assay

Human hepatic cell line HepG2 (ATCC HB-8065) was obtained from the American Type Culture Collection (Manassas, VA, USA). Transfection assays were performed as described previously [12,26]. Briefly, the cells were transfected with various expression plasmids, as well as the corresponding luciferase reporter plasmids, together with cytomegalovirus X–β-galactosidase control plasmids, using FuGENE 6 (Promega Corporation; E2691; Madison, WI, USA). The cells were then incubated with the corresponding ligands overnight, and β-galactosidase and luciferase assays were performed as described [26]. Fold activation was calculated relative to the controls. IC_50_ values were calculated by curve fitting of data using Prism software (10.4.1). For the mammalian 2-hybrid assays, HepG2 cells were transfected with GAL4 reporter, VP16-hPXR, and coregulator plasmids including GAL4-SRC1 and GAL4-PBP [25,26]. The cells were then treated with compounds at the indicated concentrations.

### 2.4. Lipid Analysis

Plasma total cholesterol and triglyceride concentrations were determined enzymatically by colorimetric methods as described previously [26]. The lipoprotein fractions were isolated by spinning 60 μL of plasma in a TL-100 ultracentrifuge (Beckman Coulter) as described previously [26]. The cholesterol content of each supernatant and the final infranatant were measured and taken to be very low-density lipoprotein (VLDL) (<1.006 g/mL), low-density lipoprotein (LDL) (1.006 ≤ density [d] ≤ 1.063 g/mL), and high-density lipoprotein (HDL) ([d] > 1.063 g/mL) cholesterol.

### 2.5. RNA Isolation and QPCR Analysis

Total RNA was isolated from mouse tissues or cells using TRIzol Reagent (Thermo Fisher Scientific; 15596026; Waltham, MA, USA), and qPCR was performed using gene-specific primers and the SYBR Green PCR kit (Bio-Rad; 170-8886; Hercules, CA, USA) as previously described [26]. The sequences of primer sets used in this study are listed in Table 1.

### 2.6. Mouse Primary Hepatocyte and Enteroid Isolation

Mouse primary hepatocytes were isolated using an optimized protocol based on a modified two-step perfusion approach [30]. After anesthesia, the inferior vena cava was cannulated to enable retrograde perfusion, which simplifies the procedure compared with portal vein cannulation. The liver was first perfused with a calcium-chelating buffer to remove blood and disrupt cell–cell junctions, followed by perfusion with Liberase collagenase to efficiently digest the extracellular matrix. Finally, the liver was dissected, and hepatocytes were purified by Percoll-based density separation, yielding a highly viable and enriched hepatocyte population.

Murine enteroids were isolated as previously described [12,26,31]. In brief, mice were euthanized, and intestines (excluding the proximal 3 cm) were excised, rinsed, and minced. Tissues were incubated in EDTA chelation buffer at 4 °C, followed by gentle shaking in dissociation buffer to release crypts. The crypts were filtered, centrifuged, washed, and embedded in Matrigel supplemented with R-spondin 1, Noggin, and EGF. The Matrigel domes were then overlaid with enteroid medium, which was replaced every 3–4 days.

### 2.7. Statistical Analysis

All data are presented as the mean ± SEM. Individual pairwise comparisons were analyzed by 2-sample, 2-tailed Student’s *t* tests. One-way analysis of variance (ANOVA) was used when multiple comparisons were made, followed by Dunnett’s *t* test. The analyses were performed using GraphPad Prism, and *p* < 0.05 was regarded as statistically significant.

## 3. Results

### 3.1. Antipsychotic Olanzapine Activates PXR In Vitro, and Sulforaphane Inhibits Olanzapine-Mediated PXR Activation

**Figure 1:**
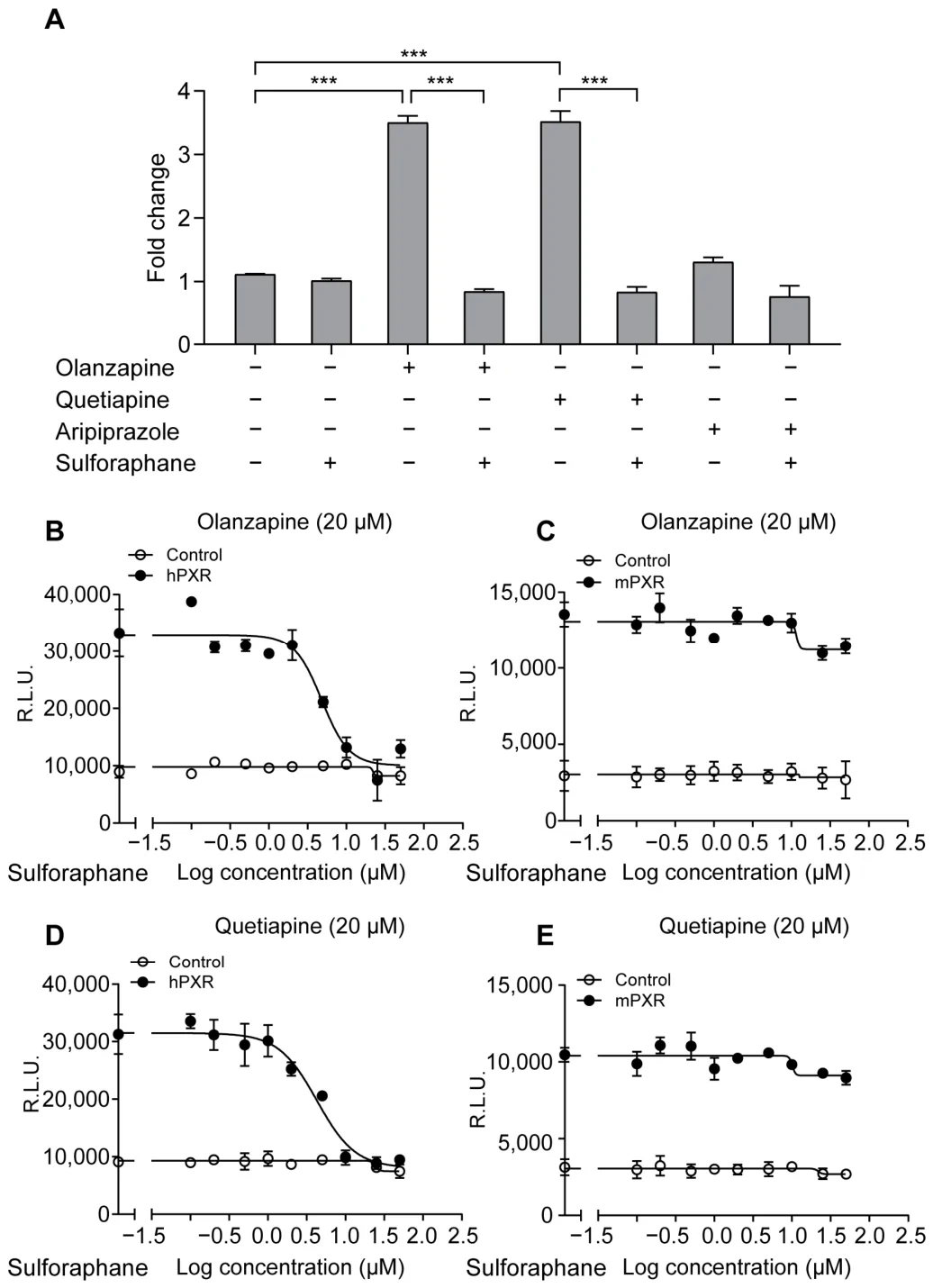
Olanzapine activates PXR, and sulforaphane inhibits antipsychotic-mediated PXR activation. (**A**) Human HepG2 cells were transfected with full-length human PXR (hPXR), together with hPXR reporter (CYP3A4-luc) and CMX–β-galactosidase plasmids. Cells were treated with DMSO control, 20 µM olanzapine, quetiapine, or aripiprazole in the absence or presence of 10 µM sulforaphane for 24 h (*n* = 3, one-way ANOVA; and *** *p*< 0.001 compared with control group). (**B**,**C**) HepG2 cells were transfected with full-length hPXR, together with hPXR reporter (CYP3A4-luc), (**B**) or full-length mouse PXR (mPXR), together with mPXR reporter ([CYP3A2]_3_-luc) (**C**) and CMX–β-galactosidase control plasmids. Cells were treated with 20 µM olanzapine with or without the indicated concentrations of sulforaphane for 24 h. (**D**,**E**) HepG2 cells were transfected with full-length hPXR, together with hPXR reporter (CYP3A4-luc), (**D**) or full-length mouse PXR (mPXR), together with mPXR reporter ([CYP3A2]_3_-luc) (**E**) and CMX–β-galactosidase control plasmids. Cells were treated with 20 µM quetiapine with indicated concentrations of sulforaphane for 24 h (*n* = 3).

We previously demonstrated that a commonly used antipsychotic, quetiapine, can activate PXR in vitro and in vivo [12]. To determine whether olanzapine can affect PXR activities, we performed transfection assays. Similarly to quetiapine, olanzapine can also activate human (h) PXR and induce hPXR-mediated CYP3A4-luciferase reporter activity in vitro (Figure 1A). Consistent with previous results [12], another antipsychotic, aripiprazole, did not affect PXR activities. We previously identified sulforaphane as a hPXR-specific antagonist [25]. Interestingly, we found that sulforaphane can efficiently inhibit olanzapine- or quetiapine-mediated hPXR activation.

### 3.2. Sulforaphane Suppresses Antipsychotic-Promoted PXR and Co-Regulator Interactions

**Figure 2:**
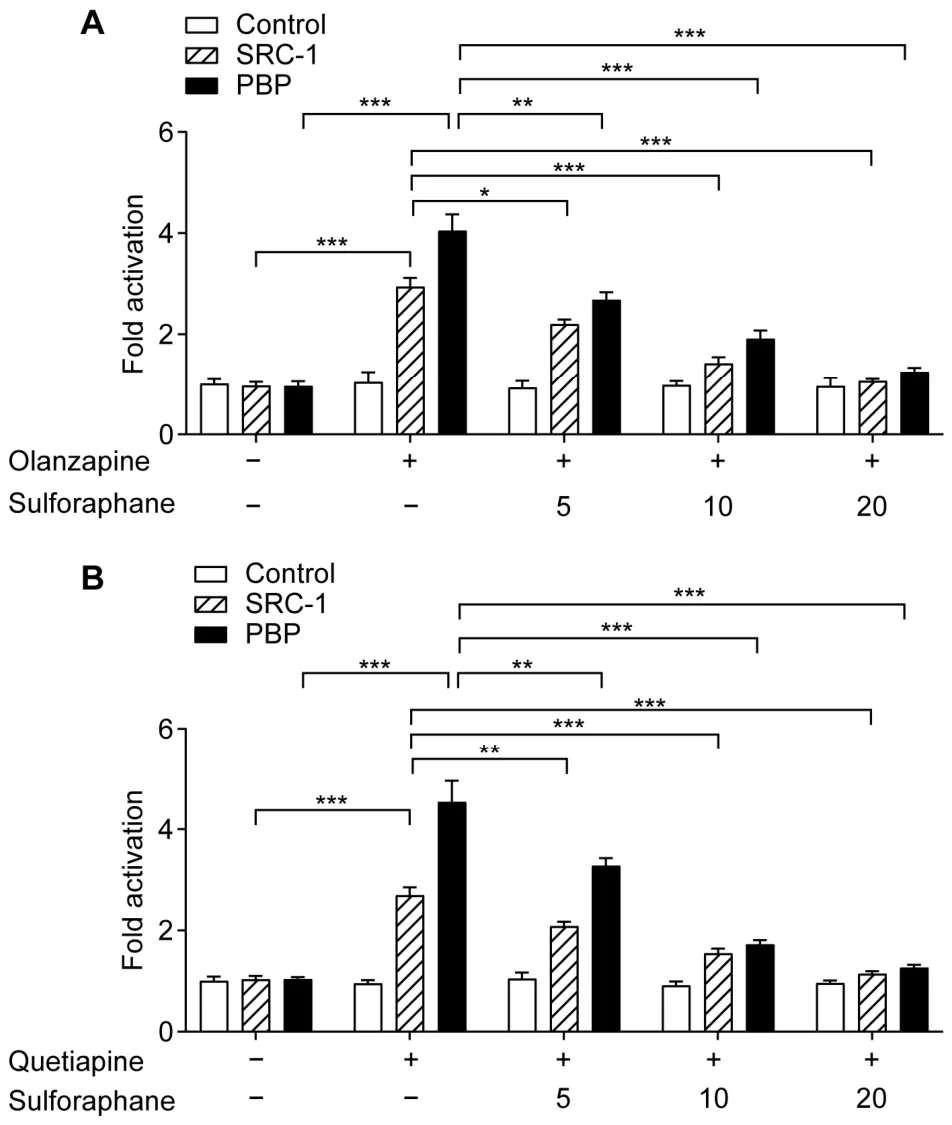
Sulforaphane inhibits olanzapine- or quetiapine-promoted PXR and coactivator interactions. HepG2 cells were transfected with a GAL4 reporter VP16-PXR vector and the expression vector for GAL4 DNA binding domain or GAL4 DNA binding domain linked to the receptor interaction domains of the indicated PXR coactivators (GAL-SRC1 and GAL-PBP). (**A**,**B**) Cells were then treated with control medium or medium containing 20 µM olanzapine (**A**) or quetiapine (**B**) in the absence or presence of sulforaphane at indicated concentrations for 24 h. (*n* = 3, one-way ANOVA; * *p* < 0.05, ** *p* < 0.01 and *** *p* < 0.001 compared with the control group).

Nuclear receptor co-regulators are required for regulation of nuclear receptor activation [26], and we previously showed that quetiapine promoted the specific interactions between PXR and the co-activators [12]. We next investigated whether sulforaphane can affect olanzapine or quetiapine-mediated PXR and co-regulator interactions (Figure 2A,B). As expected, both olanzapine and quetiapine promoted the specific interactions between PXR and coactivators steroid receptor coactivator-1 (SRC-1) and PPAR binding protein (PBP) (Figure 2A,B). However, sulforaphane attenuated olanzapine or quetiapine-stimulated interactions between PXR and co-activators in a dose-dependent manner (Figure 2A,B). Thus, sulforaphane suppresses olanzapine- or quetiapine-promoted PXR and co-activator interactions, thereby inhibiting antipsychotic-mediated PXR activation.

### 3.3. Sulforaphane Inhibits Antipsychotic-Stimulated PXR Target Gene Expression in Primary Hepatocytes and Intestinal Enteroids

**Figure 3:**
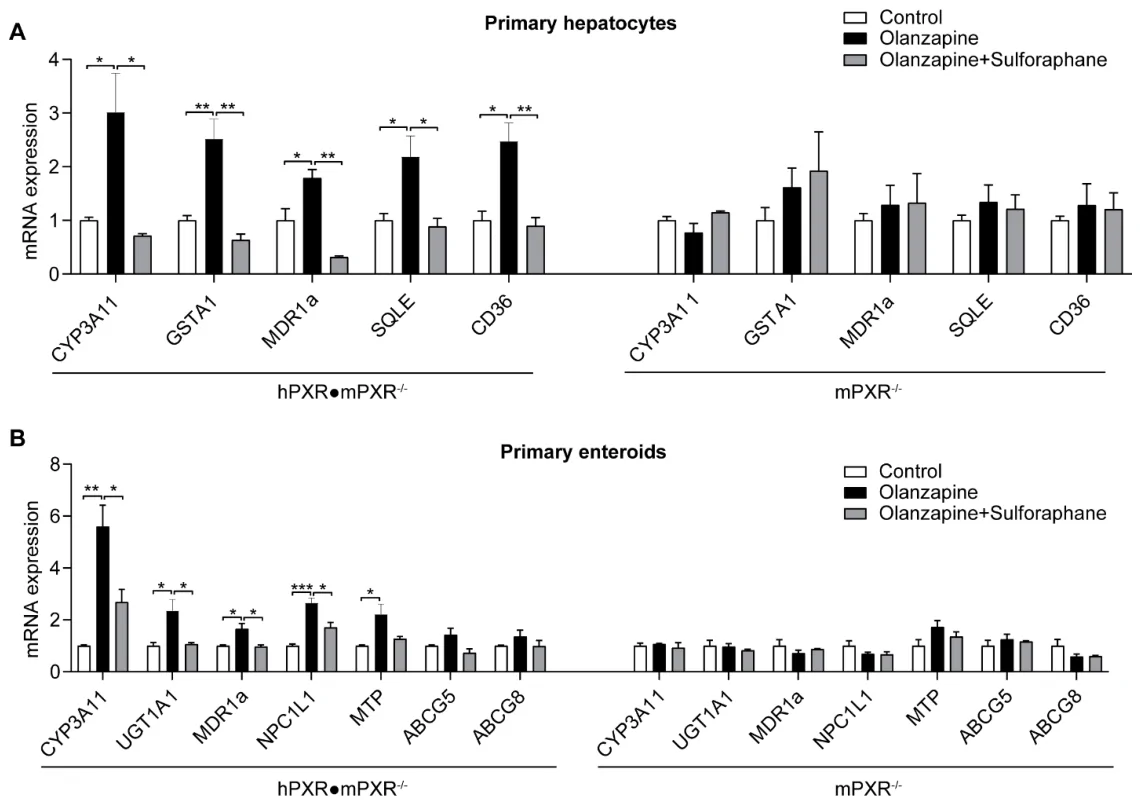
Sulforaphane inhibits antipsychotic-stimulated PXR target gene expression in primary hepatocytes and intestinal enteroids. (**A**) Primary hepatocytes isolated from hPXR●mPXR^-/-^ or mPXR^-/-^ mice were treated with DMSO control, 20 μM olanzapine or olanzapine in the absence or presence of 10 μM sulforaphane for 24 h. The expression levels of indicated genes were analyzed by QPCR (*n* = 3, one-way ANOVA; * *p* < 0.05, ** *p* < 0.01 and *** *p* < 0.001). (**B**) Primary enteroids isolated from hPXR●mPXR^-/-^ or mPXR^-/-^ mice were treated with DMSO control, 20 μM olanzapine or olanzapine in the absence or presence of 10 μM sulforaphane for 24 h. The expression levels of indicated genes were analyzed by QPCR (*n* = 3, one-way ANOVA; * *p* < 0.05, ** *p* < 0.01 and *** *p* < 0.001).

PXR is expressed at high levels in the liver and intestine and regulates key genes mediating xenobiotic and lipid metabolism in those organs [13,16]. To further evaluate the impact of sulforaphane on olanzapine-induced PXR activity, primary hepatocytes were isolated from PXR-humanized mice (hPXR●mPXR^-/-^) and mPXR knockout mice (mPXR^-/-^) and were treated with olanzapine with or without sulforaphane. The expression levels of known PXR target genes involved in xenobiotic and lipid metabolism were analyzed. Olanzapine treatment not only induced the expression of well-studied xenobiotic metabolism-related genes, including CYP3A11 and MDR1a, but also stimulated the expression of key hepatic lipogenic genes SQLE and CD36 in primary hepatocytes from hPXR●mPXR^-/-^ mice but not from mPXR^-/-^ mice (Figure 3A). As expected, sulforaphane treatment reduced olanzapine-induced expression of PXR target genes in primary hepatocytes in the presence of hPXR (Figure 3A). In addition to primary liver cells, we also isolated primary intestinal enteroids from the intestine of those mice. Consistent with primary hepatocyte results, olanzapine significantly increased the expression levels of PXR target genes mediating xenobiotic metabolism (e.g., CYP3A11 and MDR1a) and lipid homeostasis (e.g., Niemann-Pick C1-Like 1 [NPC1L1] and microsomal triglyceride transfer protein [MTP]) in the enteroids isolated from hPXR●mPXR^-/-^ mice but not mPXR^-/-^ mice (Figure 3B). Consistent with previous studies [32], olanzapine-mediated PXR activation did not affect the expression of cholesterol efflux transporters ABCG5 and ABCG8 (Figure 3B). Sulforaphane also suppressed olanzapine-induced gene expression in enteroids expressing hPXR. Taken together, these results suggest that olanzapine activates PXR signaling in primary cells, whereas sulforaphane attenuates olanzapine-stimulated expression of xenobiotic and lipid metabolism-related genes in the presence of human PXR.

### 3.4. Olanzapine Activates Intestinal PXR Signaling In Vivo

**Figure 4:**
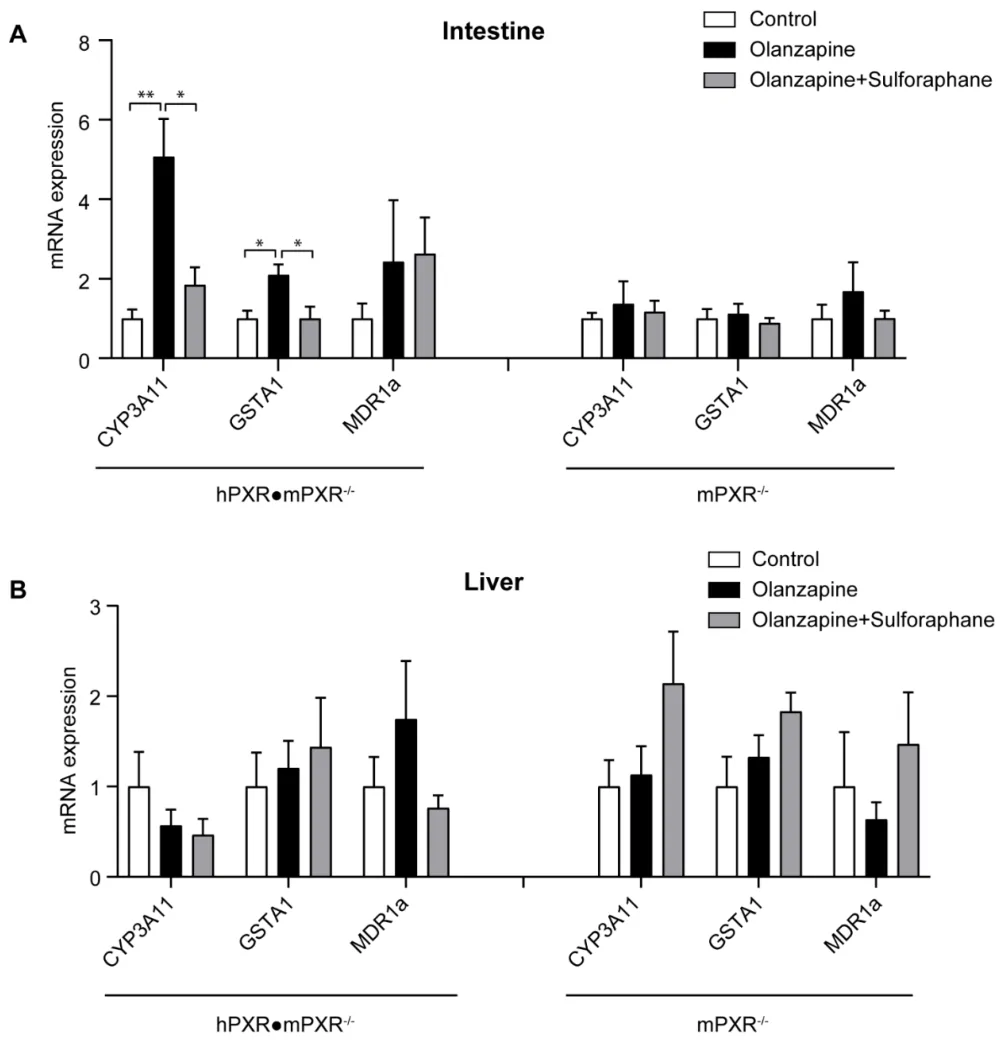
Sulforaphane attenuates olanzapine-induced PXR target gene expression in the intestine but not the liver of PXR-humanized mice. Eight-week-old male hPXR●mPXR^-/-^ mice or mPXR^-/-^ littermates were treated with vehicle control, 10 mg/kg BW/day of olanzapine, or 10 mg/kg BW/day olanzapine and sulforaphane by oral gavage for 7 days. The expression levels of indicated PXR target genes in the intestine (**A**) and liver (**B**) were analyzed by QPCR (*n* = 4–5, one-way ANOVA; * *p* < 0.05 and ** *p* < 0.01).

To further investigate the effects of olanzapine and sulforaphane on PXR signaling in vivo, hPXR●mPXR^-/-^ and mPXR^-/-^ littermates were treated with vehicle, 10 mg/kg body weight olanzapine, or 10 mg/kg body weight olanzapine and sulforaphane daily by oral gavage for 7 days. Interestingly, olanzapine treatment significantly increased the expression levels of known PXR target genes including CYP3A11, GSTA1 and MDR1a in the intestine (Figure 4A) but not in the liver (Figure 4B) in hPXR●mPXR^-/-^ mice. In contrast, olanzapine had no effect on the expression of these genes in mPXR^-/-^ mice (Figure 4). Consistent with in vitro studies, co-treatment with sulforaphane markedly suppressed olanzapine-induced PXR target gene expression in the intestine. These findings indicate that olanzapine activates intestinal but not hepatic PXR signaling in vivo.

### 3.5. Exposure to Olanzapine Leads to Elevated Cholesterol Levels in PXR-Humanized Mice, but Sulforaphane Inhibits Olanzapine-Elicited Hypercholesterolemia

**Figure 5:**
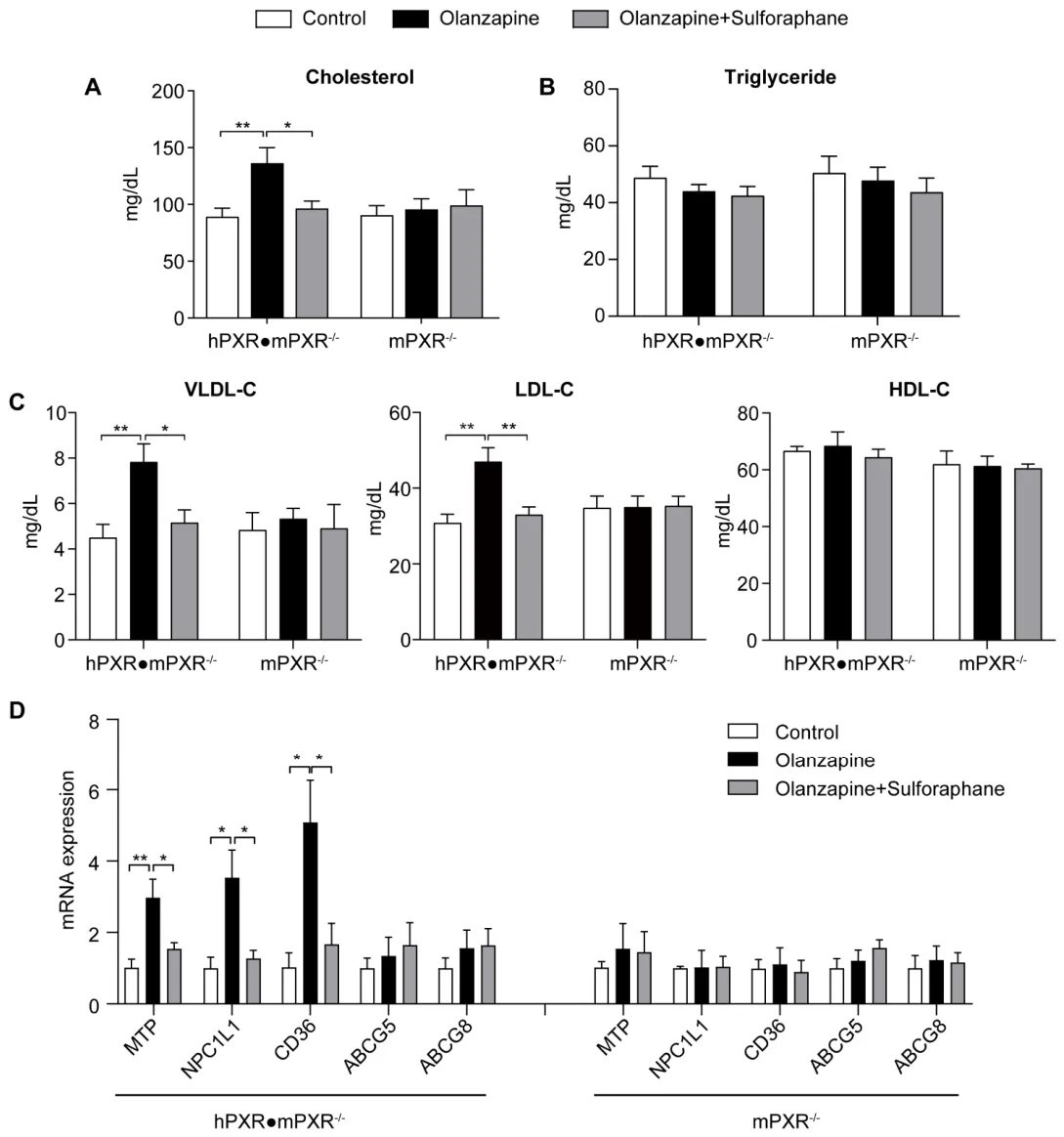
Sulforaphane attenuates olanzapine-elicited hypercholesterolemia in PXR-humanized mice. Eight-week-old male hPXR●mPXR^-/-^ mice or mPXR^-/-^ littermates were treated with vehicle control, 10 mg/kg BW/day of olanzapine, or 10 mg/kg BW/day olanzapine and sulforaphane by oral gavage for 7 days. (**A**,**B**) Plasma total cholesterol (**A**) and triglyceride (**B**) levels were measured (*n* = 4–5, two-way ANOVA; * *p* < 0.05 and ** *p* < 0.01). (**C**) Lipoprotein fractions (VLDL, LDL, and HDL) were isolated, and the cholesterol levels of each fraction were measured. (*n* = 4–5, two-way ANOVA; * *p* < 0.05 and ** *p* < 0.01). (**D**) The expression levels of indicated genes in the intestine were analyzed by QPCR (*n* = 4–5, one-way ANOVA; * *p* < 0.05 and ** *p* < 0.01).

We previously demonstrated that activation of intestinal PXR signaling by various ligands can lead to elevated circulating cholesterol levels in mice [12]. We next analyzed plasma lipid levels in the mice exposed to olanzapine and sulforaphane. Indeed, we found that olanzapine treatment led to elevated plasma total cholesterol but not triglyceride levels in hPXR●mPXR^-/-^ mice (Figure 5A,B). The increased cholesterol levels were mainly due to elevated atherogenic VLDL and LDL cholesterol levels, as HDL cholesterol levels were not affected by olanzapine treatment in those mice (Figure 5C). In addition, olanzapine treatment did not affect plasma cholesterol levels in mPXR^-/-^ mice, which suggest that PXR signaling contributed to olanzapine’s dyslipidemic effects. Intriguingly, sulforaphane treatment was able to efficiently abolish the impact of olanzapine exposure on plasma cholesterol levels (Figure 5A,C). Previous studies demonstrated that PXR can regulate several key intestinal lipogenic genes, such as NPC1L1 and MTP, which are essential for intestinal lipid absorption and lipoprotein assembly [12]. Consistent with ex vivo enteroid results (Figure 3B), olanzapine exposure significantly increased the intestinal expression of those key lipogenic genes, including NPC1L1 and MTP, in hPXR●mPXR^-/-^ mice but not in mPXR^-/-^ littermates (Figure 5D). Co-treatment with sulforaphane inhibited the olanzapine-stimulated intestinal lipogenic gene expression in those PXR-humanized mice. Collectively, these findings suggest that olanzapine treatment promotes hypercholesterolemia through activation of intestinal PXR signaling and that PXR antagonist sulforaphane may have therapeutic potential to prevent antipsychotic-elicited hyperlipidemia.

## 4. Discussion

Olanzapine is a widely prescribed atypical antipsychotic; however, its clinical utility is limited by well-documented adverse effects, including hyperlipidemia, weight gain, insulin resistance, and abnormal blood glucose levels [5,33,34]. Despite extensive clinical observations, the underlying mechanisms of these adverse effects remain poorly understood. In this study, we demonstrated that olanzapine is a potent agonist of nuclear receptor PXR, a key regulator of xenobiotic metabolism [14]. We found that olanzapine activated PXR and promoted PXR–co-activator interactions in vitro. Olanzapine exposure stimulated the expression of the known PXR target genes in primary hepatocytes and intestinal enteroids. In vivo studies then confirmed that treatment with olanzapine activated PXR signaling in the intestinal of PXR-humanized mice. Interestingly, we found that PXR-humanized mice treated with olanzapine exhibited higher circulating cholesterol levels, but PXR knockout littermates did not develop hyperlipidemia, suggesting that the effects of olanzapine on lipid levels were mediated by PXR signaling. Indeed, olanzapine-mediated PXR activation regulated several key intestinal genes mediating lipid homeostasis in PXR-humanized mice, likely contributing to olanzapine’s dyslipidemic properties. We previously identified dietary isothiocyanate sulforaphane as the specific antagonist of human PXR [25]. Sulforaphane can also inhibit olanzapine-mediated PXR activation in vitro and in vivo. Moreover, sulforaphane treatment also protected mice from olanzapine-elicited dyslipidemia. Our findings provide mechanistic insight for understanding the impact of antipsychotic olanzapine on cardiovascular disease and demonstrate the therapeutic potential of PXR antagonists in mitigating the adverse effects of PXR agonistic drugs in humans.

The function of PXR in regulating xenobiotic metabolism has been well established. Recent studies from us and others have revealed novel functions of PXR in regulating lipid homeostasis and atherosclerosis development [12,13,17,18,19,20,21,22,23,35,36]. For example, we and others demonstrated that ligand-mediated PXR activation elevates plasma total cholesterol levels and atherogenic lipoproteins such as LDL levels in mice [12,26,37]. However, the detailed mechanisms through which PXR signaling regulates lipid homeostasis are not completely understood. PXR is expressed at high levels in the liver and intestine, which are two essential organs mediating whole-body lipid homeostasis. PXR has been shown to regulate the expression of multiple hepatic lipogenic genes, including CD36, squalene epoxidase, stearoyl-CoA desaturase-1, lipin-1, Insig-1, and Proprotein convertase subtilisin/kexin type 9 (PCSK9) [13,17,18,19,20,21,22,23,38].

Olanzapine was able to activate PXR and stimulated the expression of its target genes in primary hepatocytes in vitro. However, short-term treatment with olanzapine did not affect the known PXR target-gene expression in the liver of PXR-humanized mice. Previous studies have identified several tissue-specific PXR ligands, including rifaximin [39,40]. Rifaximin is a nonsynthetic antibiotic that is a potent intestine-specific PXR ligand [39]. After oral treatment, rifaximin is accumulated in the intestine, which likely leads to the tissue-specific effects of rifaximin on intestinal PXR signaling. In the current study, the relatively low dose and short treatment duration may have led to low olanzapine concentrations in the liver. It is also plausible that olanzapine can be rapidly metabolized by hepatocytes in vivo. Future studies are warranted to examine the internal concentrations of olanzapine and its metabolites and investigate the mechanisms underlying the tissue-specific effects of olanzapine and other PXR ligands. In addition to the liver, recent studies identified several novel transcriptional targets of PXR in the intestine, including NPC1L1 and MTP, which play key roles in regulating intestinal lipid absorption and lipoprotein assembly and secretion [12,26]. For example, we identified PXR binding sites located in the promoters of NPC1L1 and MTP and confirmed that PXR can directly regulate the transcription of NPC1L1 and MTP [12,26]. Activation of PXR by relevant agonists stimulated cholesterol uptake by enterocytes or enteroids and increased cholesterol absorption in mice [12,26]. Indeed, another widely used antipsychotic, quetiapine, activated intestinal PXR signaling to stimulate NPC1L1 and MTP expression, leading to increased intestinal cholesterol absorption and hypercholesterolemia [12]. In the current study, we found that olanzapine also activated PXR signaling and upregulated key intestinal lipogenic genes, including NPC1L1 and MTP, in primary enteroids and in vivo. The functions of those genes in lipid homeostasis have been well studied. For example, NPC1L1 is a transmembrane protein critical for the intestinal absorption of dietary and biliary cholesterol, thereby regulating whole-body cholesterol homeostasis [41,42,43,44,45,46]. MTP is an essential ER-resident protein that facilitates the assembly and secretion of apolipoprotein B-containing lipoproteins by transferring triglycerides, cholesterol esters, and phospholipids to nascent lipoproteins, thereby playing a central role in lipid transport and lipoprotein metabolism [47,48,49,50]. In addition to NPC1L1 and MTP, olanzapine treatment also elevated the intestinal expression of CD36, a multifunctional transmembrane receptor that plays an important role in regulating intestinal cholesterol uptake [45,51,52]. Thus, PXR-mediated upregulation of those key intestinal lipogenic genes may lead to increased cholesterol absorption and contribute to olanzapine-stimulated elevation of circuiting cholesterol levels.

For the treatment of bipolar disorder, the standard clinical dose of olanzapine is 10 mg per day for patients [53]. For mouse treatment studies, the drug doses were calculated based on an interspecies scaling factor of 12.3 between mice and humans [28,29]. Consequently, mouse treatment studies usually require 12.3 times more drug in order to be comparable to the dose in humans. Thus, the 10 mg/kg/day olanzapine dose we used in this study is below the human dose range [28,29]. In addition, the 1-week treatment duration was selected based on previous studies on PXR ligand-induced dyslipidemia [12,26]. For example, we showed that 1-week exposure to 10 mg/kg/day quetiapine was sufficient to affect lipid homeostasis and induce dyslipidemia in mouse models [12]. Thus, we used the same dose and treatment duration for the current olanzapine study to keep it consistent. Although olanzapine has documented adverse effects, the recent approval of olanzapine–samidorphan combination therapy for the treatment of schizophrenia and bipolar disorder has demonstrated efficacy in partially mitigating olanzapine-induced weight gain and metabolic disorders [33,54]. This also highlights the potential of combining olanzapine with other agents as a strategy to mitigate its adverse cardiometabolic effects. Since our results suggest that olanzapine-mediated PXR activation may contribute to its dyslipidemic effects, the targeting of PXR signaling by appropriate inhibitors may prevent or reduce the adverse effects of olanzapine on lipid homeostasis. We previously identified sulforaphane as the first naturally occurring antagonist for human PXR [25]. SFN is among the most biologically active phytochemicals in the human diet, found at high concentrations in cruciferous vegetable broccoli and broccoli sprouts. Sulforaphane has attracted considerable attention as a potential therapeutic candidate for a wide range of diseases. It has been demonstrated to have anti-oxidant, anti-inflammatory, neuroprotective, chemopreventive, and antidiabetic effects [55,56,57,58,59,60,61,62,63]. Sulforaphane has been shown to exert these effects through multifactorial mechanisms [25,64,65,66]. We previously found that sulforaphane can bind directly to human PXR, inhibit PXR-coactivator recruitment, and efficiently repress PXR activity [25]. In the current study, we also found that sulforaphane efficiently blocked olanzapine and quetiapine-mediated human PXR activation. Interestingly, sulforaphane is a specific antagonist of human PXR without inhibitory effects on rodent PXR (mouse and rat) [25]. Consistently, we also confirmed that sulforaphane only inhibited olanzapine- or quetiapine-induced hPXR activity but not mPXR activity. The in vivo effects of sulforaphane on PXR activities are not well understood, and previous studies showed inconsistent results [67,68,69,70]. Since sulforaphane is a hPXR-specific ligand, we conducted animal studies using PXR-humanized mice and their mPXR^-/-^ littermates. We found that exposure to sulforaphane efficiently attenuated olanzapine-induced intestinal PXR target-gene expression and hypercholesterolemia in PXR-humanized mice but not in mPXR^-/-^ mice. Our results indicate a potentially novel approach to combat the atherogenic effects of PXR-activating antipsychotics and other drugs.

## 5. Conclusions

In summary, we identified widely used antipsychotic olanzapine as a potent PXR agonist that stimulated key intestinal lipogenic gene expression in primary cells and in vivo. Exposure to olanzapine led to elevated circulating total cholesterol and atherogenic VLDL and LDL cholesterol levels in PXR-humanized mice. We also explored the therapeutic potential of a previously identified human PXR antagonist, sulforaphane, and found that it can efficiently block olanzapine-elicited PXR activation in vitro and in vivo and attenuate olanzapine-elicited hypercholesterolemia. Our results not only provide important insights for understanding the mechanisms through which antipsychotic olanzapine and other related drugs induce dyslipidemia but also provide an innovative approach to mitigate the adverse effects of PXR agonistic drugs on cardiovascular health.

## Figures and Tables

**Table 1:** Primer sequences used for QPCR.

Genes	Forward Primer Sequences (5′-3′)	Reverse Primer Sequences (5′-3′)
mCYP3A11	CAGCTTGGTGCTCCTCTACC	TCAAACAACCCCCATGTTTT
mUGT1A1	GCTTCTTCCGTACCTTCTGTTG	GCTGCTGAATAACTCCAAGCAT
mMDR1a	CCCCCGAGATTGACAGCTAC	ACTCCACTAAATTGCACATTTCCTTC
mGSTA1	CAG CCT GGC AGC CAG AGA	TCTGTGGCTCCATCAATGCA
mCD36	CAGTCGGAGACATGCT	CTCGGGGTCCTGAGTT
mNPC1L1	TTGCCTTGACCTCTGGCTTAG	AGGGCGGATGAATCTGTGC
mMTP	TGAGCGGCTATACAAGCTCAC	CTGGAAGATGCTCTTCTCGC
mSQLE	ATAAGAAATGCGGGGATGTCAC	ATATCCGAGAAGGCAGCGAAC
mABCG5	TGCCCATTCCTTTAAAAATCC	GATGAACTGGACCCCTTGG
mAGCG8	GTAGCTGATGCCGATGACAA	GGGGCTGATGCAGATTCA
mGAPDH	AACTTTGGCATTGTGGAAGG	GGATGCAGGGATGATGTTCT

## Data Availability

Data that supports the plots within this publication and other findings of this study are available from the corresponding author upon reasonable request.
